# Supplementary material for: A reversible mitochondrial complex I thiol switch mediates hypoxic avoidance behavior in C. elegans
Source: Nat Commun. 2022 May 3;13:2403. doi: 10.1038/s41467-022-30169-y (PMC9064984; doi:10.1038/s41467-022-30169-y)
Supplement: Supplementary file 1 — Supplementary Information [file 41467_2022_30169_MOESM1_ESM.pdf]

A reversible mitochondrial complex I thiol switch mediates hypoxic avoidance behavior in *C. elegans*

John O. Onukwufor, M. Arsalan Farooqi, Anežka Vodičková, Shon A. Koren, Aksana Baldzizhar, Brandon J. Berry, Gisela Beutner, George A Porter Jr, Vsevolod Belousov, Alan Grossfield, and Andrew P. Wojtovich

Supplementary Fig. 1.

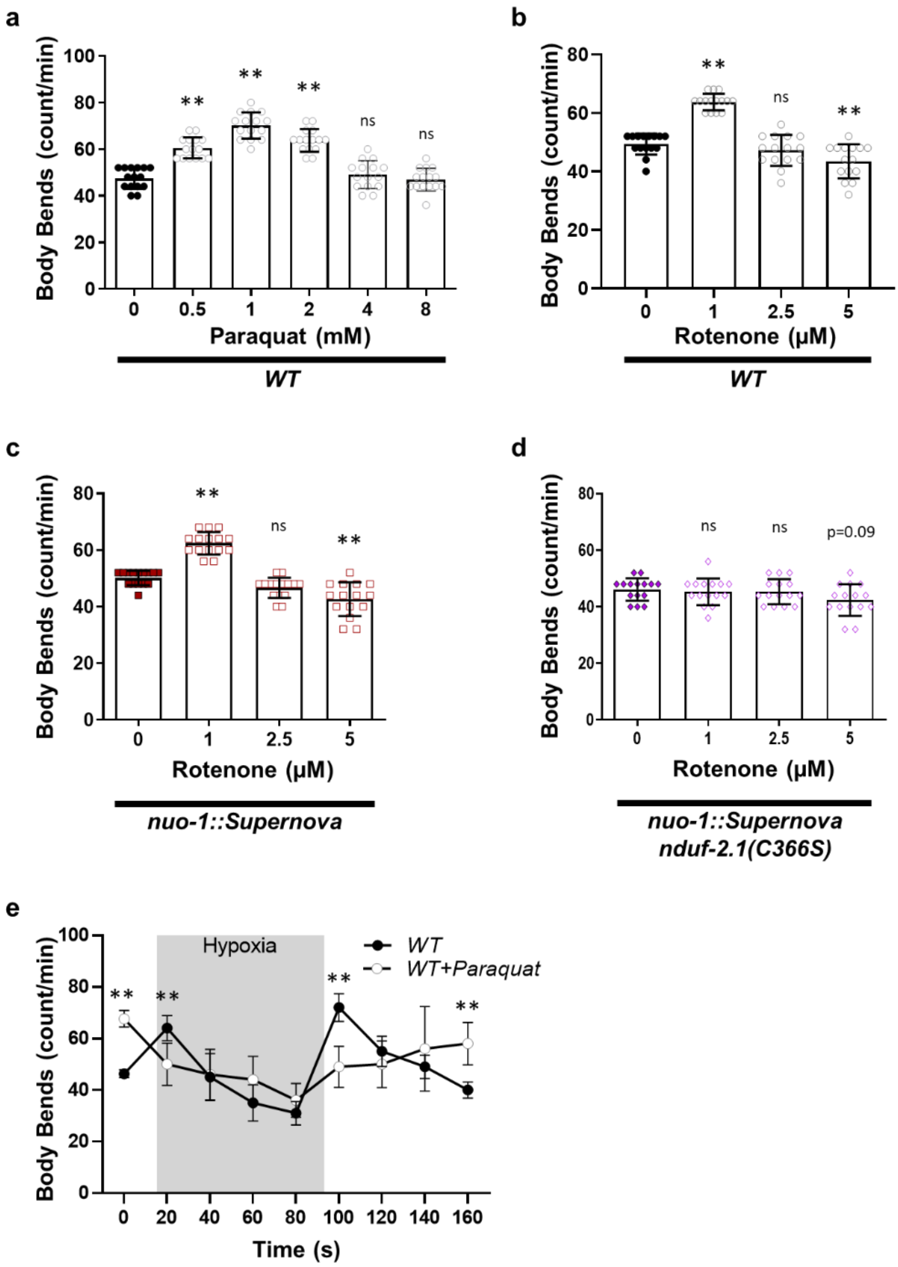

Supplementary Figure 1. Dose-response of complex I ROS inhibitor-mediated behavioral changes. a Wild-type L4 worms were treated with paraquat (0, 0.5, 1, 2, 4 or 8 mM) 24 hr prior. **b-d** (**b**) Wild-type L4, (**c**) *nuo-1::SuperNova*, and (**d**) *nuo-1::SuperNova* + *nduf-2.1-Cys366S* worms were treated with rotenone (0, 1, 2.5 or 5 μM) 24 hr prior. Body bends were scored on unseeded plates. Data are mean ± SD. N = 15 independent animals across 3 technical

replicates, **p<0.01 (One-way ANOVA, Dunnett's multiple comparisons). The 0 and 1 µM data points from this dose response Figure are presented in Figures 1B-C and 6E and reproduced here for comparison. **e** *C. elegans* increase locomotion in response to changes in oxygen concentration. Staged wild-type (N2 Bristol) L4 body bends were scored on unseeded plates in response to changes in oxygen. Baseline was recorded, and worms were then subjected to hypoxia and reoxygenation. Where indicated, worms were grown on plates containing paraquat (1 mM) 24 hr prior. Data are mean ± SD. N = 15 independent animals across 3 technical replicates, **p<0.01 vs WT (Two-way ANOVA, Sidak's multiple comparisons).

Supplementary Fig. 2.

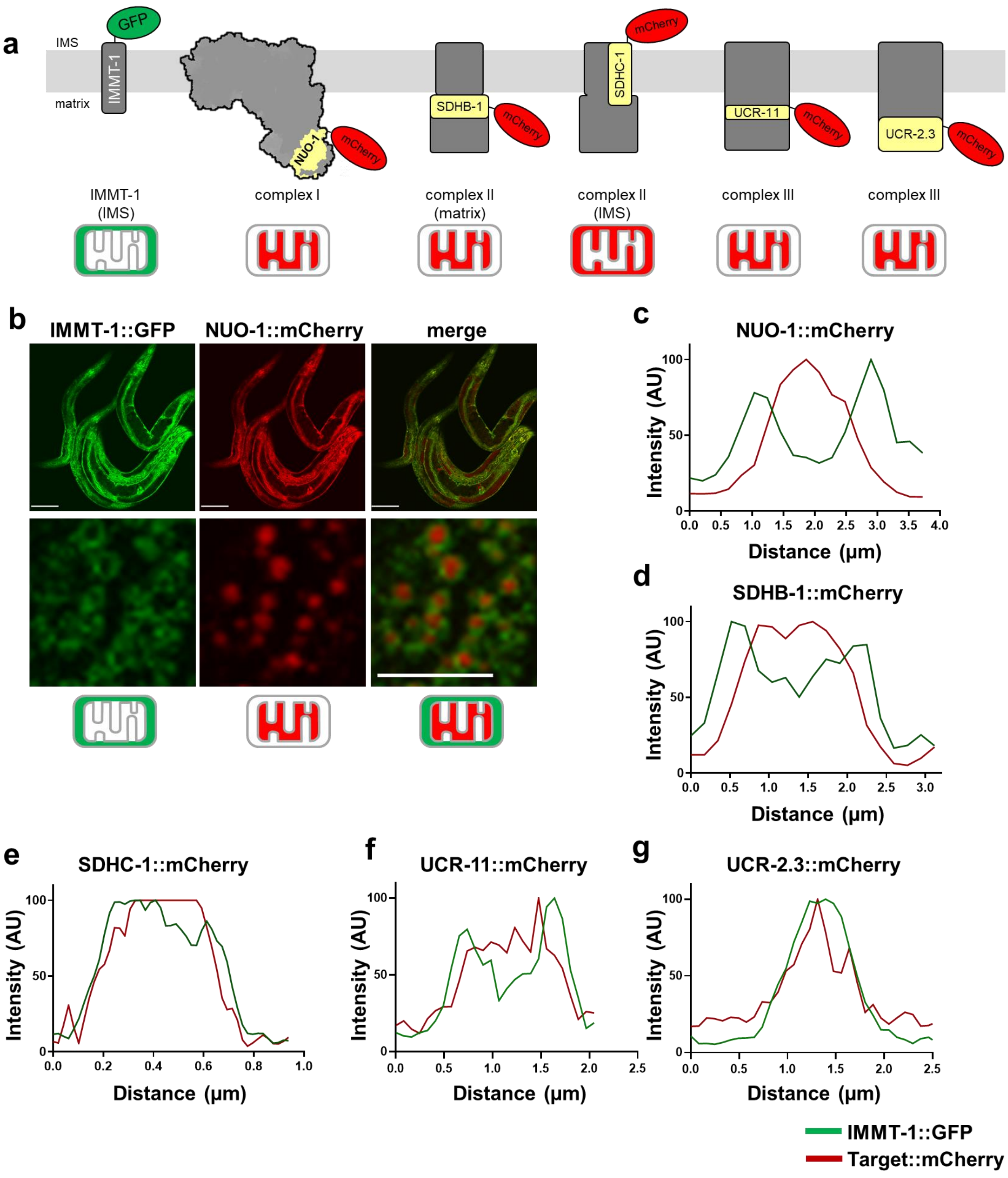

Supplementary Figure 2. Line scans of fluorescent protein in mitochondria. a Schematic of mitochondrial electron transport chain complexes and the location of the fluorescent tag. **b** Representative image of *nuo-1::mCherry* fusion proteins. Images (>3) were taken and quantified in Figure 2. Compartmentalization of the fluorescent protein was assessed using IMMT:GFP, which aids in cristae formation and is restricted to outer membrane and inner membrane contact sites. Scale bar 100 and 10 µm. **c-g** Line scans of fluorescent protein signals across a single hypodermal mitochondrion for (**c**) *nuo-1::mCherry*, (**d**) *sdhb-1::mCherry*, (**e**) *sdhc-1::mCherry,* (**f**) *ucr-11::mCherry* and (**g**) *ucr-2.3::mCherry* lines. Quantification of line scans for *sdhb-1::mCherry* (RFP matrix), *sdhc-1::mCherry* (RFP IMS), and *nuo-1::Supernova* are presented for in Figure 2.

Supplementary Fig. 3.

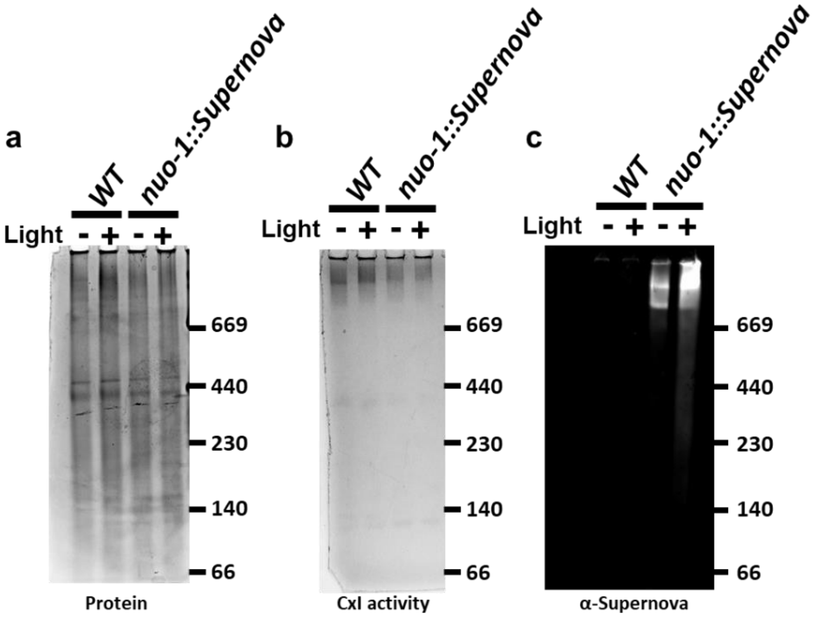

Supplementary Figure 3. Clear native electrophoresis of mitochondria isolated from wild-type and *nuo-1::Supernova C. elegans*. Mitochondria were isolated and separated using clear native electrophoresis. Gels were either (**a**) stained with Coomassie, (**b**) subjected to an in-gel activity assay measuring complex I NADH activity, or (**c**) Western blotted for Supernova. Where indicated, samples were treated with light (GYX, 7.8 mW/mm^2) for 0 or 10 min. Western blots were repeated 6 times and quantified in supplemental Figure 9. Molecular weight markers in kDa are provided to the right of each image.

Supplementary Fig. 4.

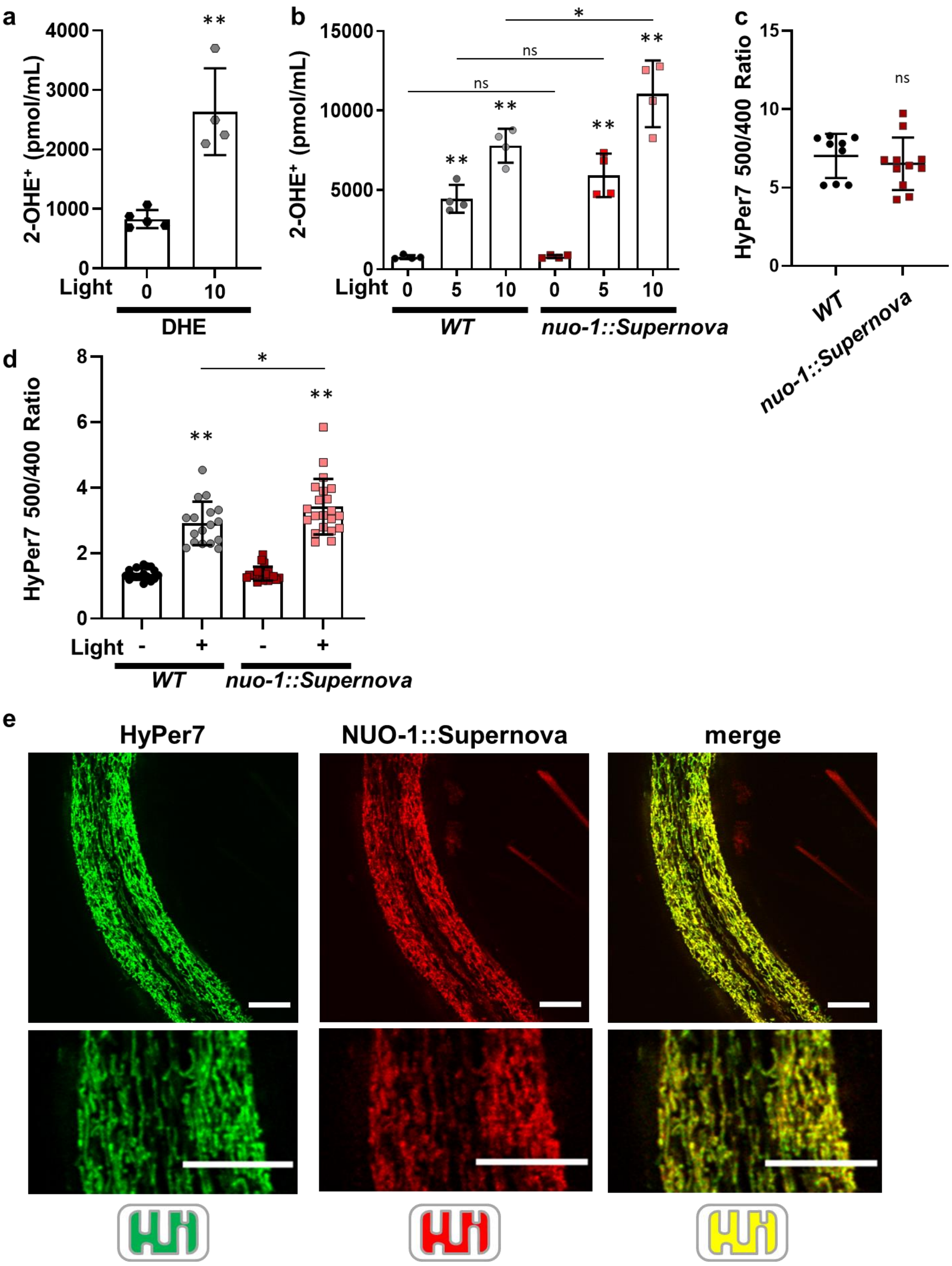

Supplementary Figure 4. Optogenetic generation of complex I ROS production. a Light-induced superoxide detection using dihydroethidium oxidation product 2-hydroxyethidium (2-OHE^+). Baseline rate of dihydroethidium (DHE) oxidation with light (GYX, 7.8 mW/mm^2) for the indicated time. 2-OHE^+ was separated from other oxidation products using HPLC. Data are mean ± SD. N = 4, 5 independent mitochondrial preparations, **$p<0.01$ (Unpaired two-tailed t-test). **b** Raw data of superoxide production by *nuo-1::Supernova* in isolated mitochondria. Mitochondria isolated from wild-type and *nuo-1::Supernova* worms were illuminated (GYX, 7.8 mW/mm^2) for the indicated time, in the presence of dihydroethidium (DHE) (100 µM) for 2-OHE^+ separation using HPLC. Data are mean ± SD. N = 4 independent mitochondrial preparations, *$p<0.05$, **$p<0.01$ (Two-way ANOVA, Tukey's multiple comparisons). Data are normalized to account for light effects on DHE and presented in Figure 2E. **c** Mitochondria matrix-targeted HyPer7 measurement in response to saturating H_2O_2. Staged L4 worms from wild-type and *nuo-1::Supernova* were screened for bright pharyngeal expression of HyPer7. Baseline expression and signal across groups was measured after addition of 1mM H_2O_2. Data are mean ± SD. N = 9, 11 independent animals across 3 technical replicates, ns = not significant (Unpaired two-tailed t-test). **d** Mitochondria matrix-targeted HyPer7 measurement in response to light. Staged L4 worms from wild-type and *nuo-1::Supernova* were screened for bright pharyngeal expression of HyPer7. Worms were then placed into glass-bottom 96-well plate containing M9 buffer. Worms were illuminated (GYX, 1.44 mW/mm^2) for 2 mins. Data are mean ± SD. N = 17, 17, 21, 21 independent animals across 3 technical replicates, *$p<0.05$, **$p<0.01$ (Two-way ANOVA, Tukey's multiple comparisons) vs no light and as indicated. Data are raw HyPer7 ratios (500/400nm). These data are normalized to the WT genotype condition to account for the light effect and presented in Figure 2F. **e** Mitochondria matrix-localization of HyPer7. Localization of the fluorescent protein to the matrix was assessed using confocal microscopy. Image is representative of at least three independent experiments. Scale bar 10 µm.

Supplementary Fig. 5.

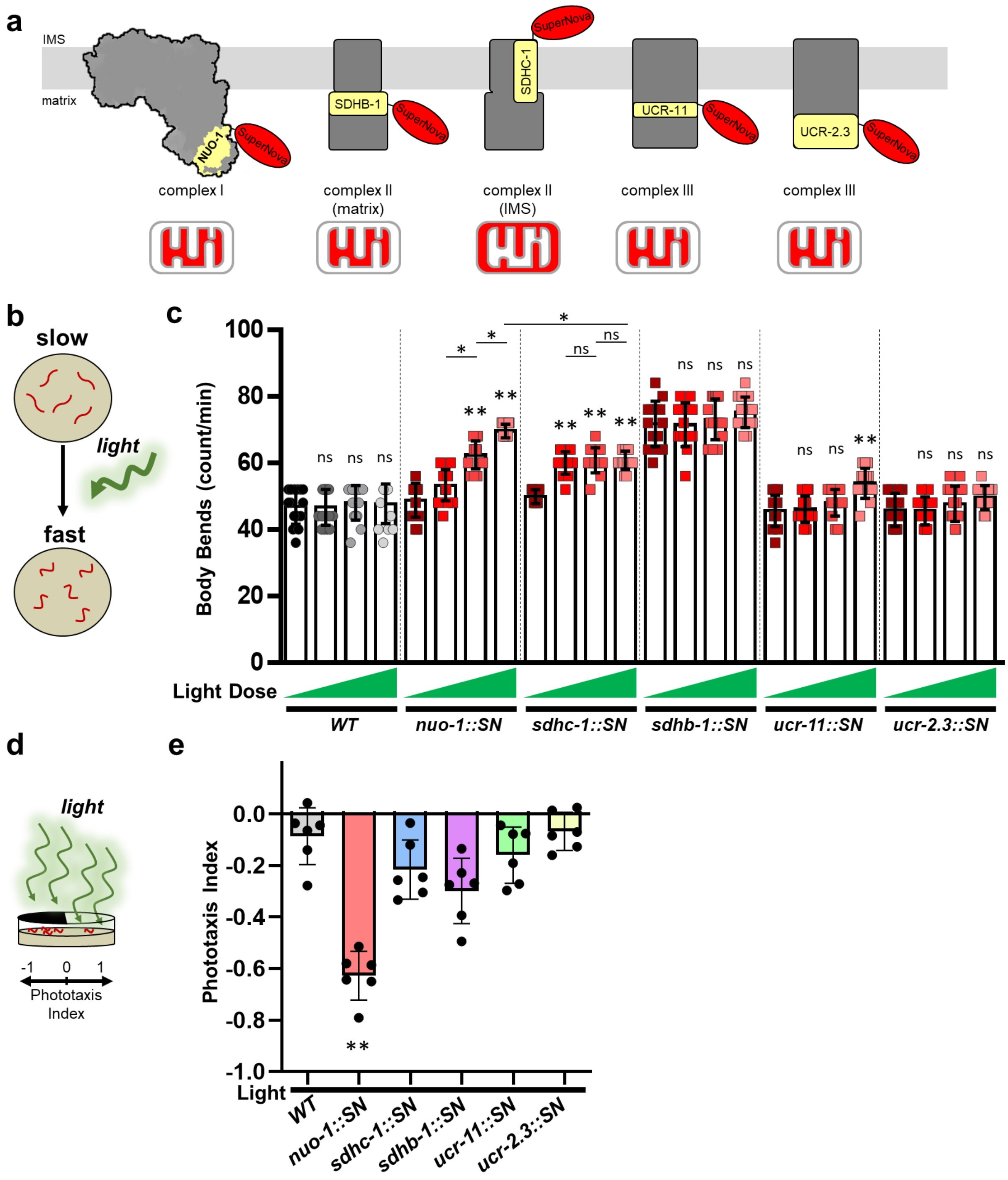

Supplementary Figure 5. Site-specific light titration of *C. elegans* locomotion in response to light. a Schematic illustration of electron transport chain and sites of light-induced ROS production. **b** Schematic illustration of the body bends experiment. **c** Site-specific light titration. Staged L4 wild-type, *nuo-1::Supernova, sdhc-1::Supernova, sdhb-1::Supernova, ucr-11::Supernova, and ucr-2.3::Supernova* worms were individually transferred to unseeded plates and body bends were scored with and without light doses (MVX, 0, 0.9, 2.5 and 5.6 mW/mm^2) for 15s. The wild-type and *nuo-1::Supernova* dose responses are shown in Figure 3 and reproduced here for comparison. Data are mean ± SD. N = 15 independent animals across 3 technical replicates, *p<0.05, **p<0.01 (Two-way ANOVA, Tukey's multiple comparisons). **d** Schematic illustration of phototaxis experimental procedure. **e** Wild-type (N2), *nuo-1::Supernova, sdhc-1::Supernova, sdhb-1::Supernova, ucr-11::Supernova and ucr-2.3::Supernova* worms were transferred to the center of the seeded plate with half of the plate shaded (dark). Worms were then illuminated (GYX, 0.78 mW/mm^2) for 120 min and the number of worms in the light and dark sections were scored and the phototaxis index was calculated. Data are mean ± SD. N = 6 independent experiments, each containing 25-80 animals, **p<0.01 vs all groups (One-way ANOVA, Tukey's multiple comparisons).

Supplementary Fig. 6.

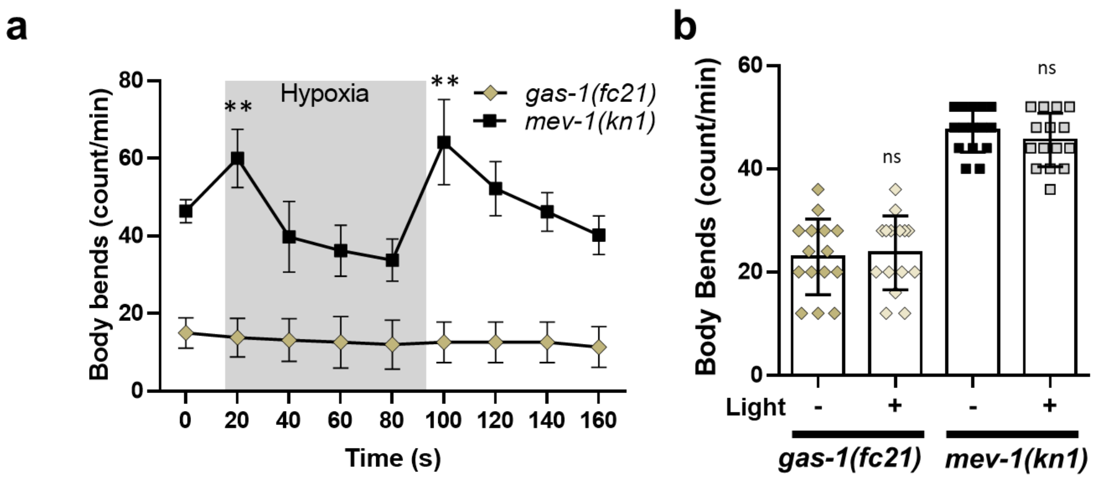

Supplementary Figure 6. Hypoxia-mediated behavioral changes in mitochondrial mutants. a *C. elegans* increase locomotion in response to changes in oxygen concentration. Staged *gas-1(fc21)* and *mev-1(kn1)* L4 body bends were scored on unseeded plates in response to changes in oxygen. Baseline was recorded, and worms were then subjected to hypoxia and reoxygenation. Data are mean ± SD. N = 15 independent animals across 3 technical replicates, **p<0.01 (Two-way ANOVA, Sidak's multiple comparisons). **b** Locomotion in response to light. *C. elegans* were acclimated to an unseeded plate and body bends were counted before and after illumination. Staged *gas-1(fc21)* and *mev-1(kn1)* L4 worms were individually transferred to unseeded plates and body bends were scored with and without light (MVX, 5.6 mW/mm^2) for 15s. Data are mean ± SD. N = 15 independent animals across 3 technical replicates, **p<0.01 vs no light (Two-way ANOVA, Sidak's multiple comparisons).

Supplementary Fig. 7.

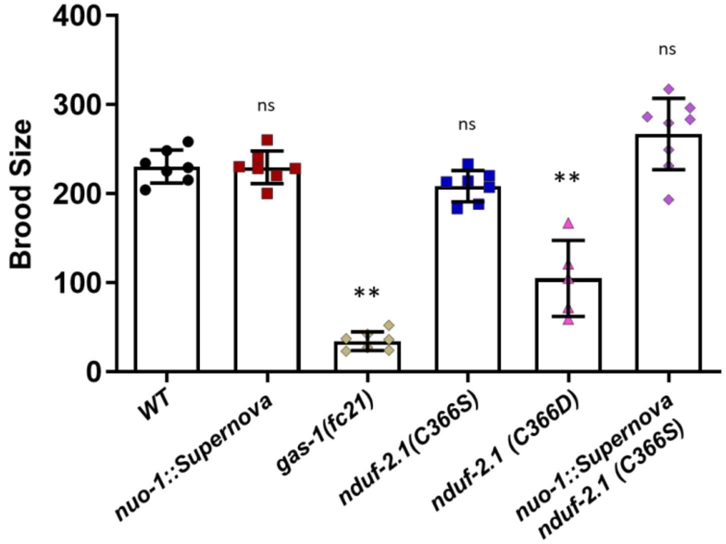

Supplementary Figure 7. Effects of Single thiol switch on brood size. Staged L4 wild-type, *nuo-1::SuperNova*, *gas-1(fc21)*, *nduf-2.1(C366S)*, *nuo-1::SuperNova* with *nduf-2.1(C366S*), and *nduf-2.1(C366D*) worms were transferred to a seeded plate every 24 hr for 8 days. The viable progeny from each plate were counted to determine the brood size. Data are mean ± SD. N = 7, 7, 7, 7, 5, 8 independent animals across 1-3 technical replicates, **p<0.01 (One-way ANOVA, Tukey's multiple comparisons).

Supplementary Fig. 8.

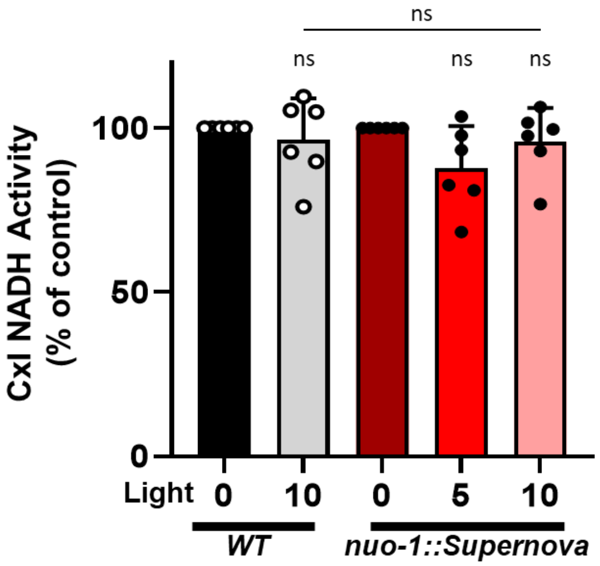

Supplementary Figure 8. Effects of light induced complex I ROS on complex I NADH dehydrogenase activity using DPI-sensitive rate of DCPIP reduction. Isolated freeze-thawed mitochondria from wild-type and *nuo-1::SuperNova* worms were exposed to light (7.8 mW/mm^2) for 0, 5 and 10 min. NADH dehydrogenase activity of complex I was measured as NADH driven DPI-sensitive rate of DCPIP reduction. Data are mean ± SD. N = 6 independent mitochondrial preparations, ns = not significant (Two-way ANOVA, Tukey's multiple comparisons).

Supplementary Fig. 9.

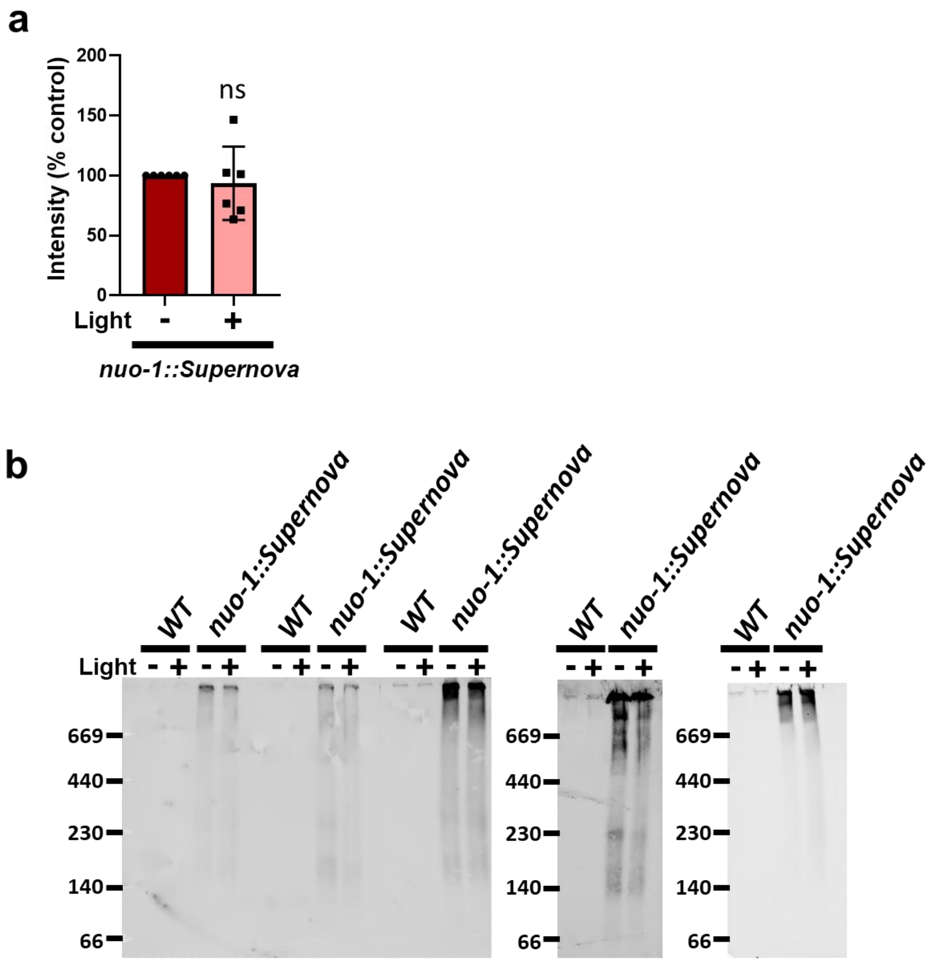

Supplementary Figure 9. NUO-1::Supernova protein levels following illumination. a Mitochondria isolated from wild-type and *nuo-1::Supernova* worm were subjected to light (GYX, 0.78 mW/mm^2) for 10 min where indicated. Samples were subjected to clear native electrophoresis and Western blotted for Supernova. Complex I was quantified and presented as a percent of the no light control. Data are mean ± SD. N = 6 separate treatments from 3 independent mitochondrial preparations. p>0.05 (one-sample T-test). **b** Western blot images used to quantify complex I in response to light. Blot containing the sixth sample is provided in Supplementary Fig. 3C. Molecular weight markers in kDa are provided to the left of each image.

Supplementary Fig. 10.

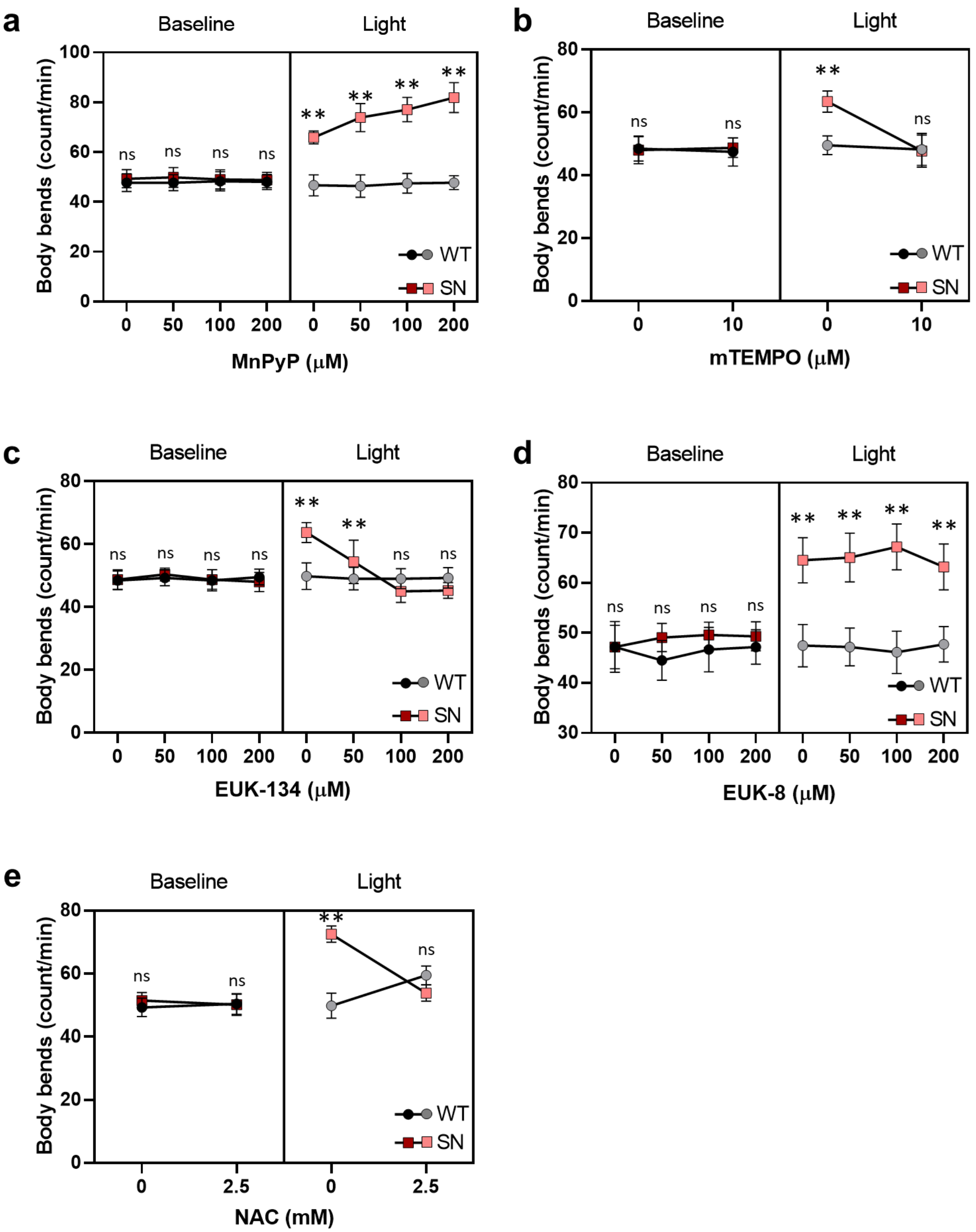

Supplementary Figure 10. Dose response of pharmacologic modulations of complex I ROS induced changes in *C. elegans* locomotion. Full dose response of staged L4 worms

wild-type and *nuo-1::SuperNova* worms to (**a**) MnPyP (0, 50, 100 and 200 µM), (**b**) mTEMPO (0 and 10 µM), (**c**) EUK-134 (0, 50, 100 and 200 µM) (**d**) EUK-8 (0, 50, 100 and 200 µM) and (**e**) NAC (0, 2.5 mM). Worms were incubated for 24 hr on a plate containing the compound. Worms were then transferred on to an unseeded plate and body bends were counted for 15 s with and without illumination (MVX, 5.6 mW/mm^2). Data are mean ± SD. N = 15 independent animals across 3 technical replicates, **p<0.01 vs wild-type (Three-way ANOVA, Tukey's multiple comparisons). *nuo-1::SuperNova* data (MnPYP, 0 and 100 µM; mTEMPO, 0 and 10 µM; EUK, 0 and 100 µM; NAC, 0 and 2.5 mM) are presented in Figure 5 and reproduced here for comparison.

Supplementary Fig. 11.

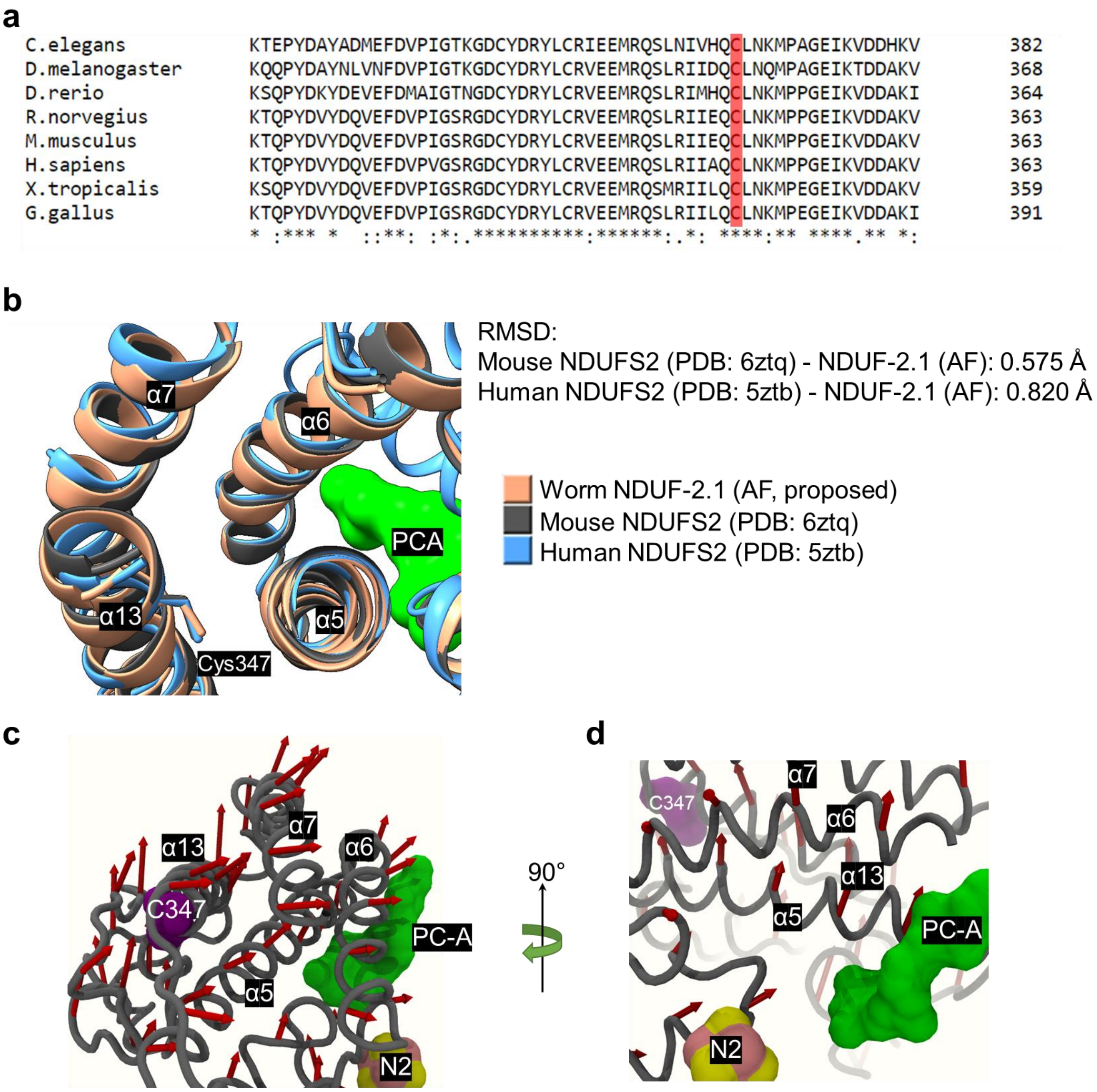

Supplementary Figure 11. In-depth comparison of NDUF-2.1 to vertebrate homologs. a Proximal protein sequence alignment (clustal omega) between *C. elegans* C366 NDUF-2.1 and vertebrate orthologs. **b** Structural comparison between solved *M. musculus* NDUFS2 and *H. sapiens* NDUFS2 and Alpha-Fold (AF) proposed structure for *C. elegans* NDUF-2.1 (ChimeraX). PDB structures, where available, are denoted in parenthesis. α-helices and piericidin A (PCA, green) are indicated. **c-d** Visualization of mode 1 flexibility from network model analysis of mouse complex I (PDB: 6ZR2) with relevant structures in NDUFS2 shown. The surface of Cys347 (mouse ortholog to *C. elegans* Cys366) is shown, internally facing adjacent helix α5. Elastic network model mode analysis, performed using ProDy[59], estimates possible movement of the alpha carbon for each residue in complex I using a harmonic model, like beads on a spring. Arrows indicate proposed direction of flexibility, scaled up for visibility, from the largest model obtained from this analysis. The roughly parallel direction of helices 5-6-7-13 moves concertedly, like a rigid body. We hypothesize that oxidation of Cys347/366 may create a steric clash within this domain and thus destabilize this helical complex and Q binding. Q-like piericidin A (PC-A) is shown in green (PDB: 6ZTQ) superimposed for visualization of the Q binding pocket, whereas the Fe-S complex N2 is colored by heteroatom.

Supplementary Table 1. *C. elegans* strains.

Strain	Genotype	Abbreviation	Source	Description
N2(Bristol)	*WT*	Wild type	*C. elegans* Genetics Center (CGC)	Wild type
APW92	*nuo-1(jbm16 [nuo-1::link::SuperNova]) II*	*nuo-1::SuperNova*	this study	SuperNova complex I fusion.
APW54	*sdhb-1(jbm12 [sdhb-1::SuperNova]) II*	*sdhb-1::SuperNova*	PMID: 30887829	SuperNova complex II fusion (matrix).
APW149	*sdhc-1(jbm34 [sdhc-1::linker::SuperNova]) III*	*sdhc-1::SuperNova*	PMID: 30887829	SuperNova complex II (Intermembrane space).
APW40	*immt-1(jbm7 [immt-1::link::GFP]) X*	*immt-1::GFP*	PMID: 30887829	GFP mitofilin fusion. Intermembrane space localization of GFP. Used for localization studies.
APW75	*sdhb-1(jbm4 [sdhb-1::mCherry]) II; immt-1(jbm7 [immt-1::link::GFP]) X* .	*immt-1::GFP; sdhb-1::cherry*	PMID: 30887829	Ubiquitously expressed mCherry complex II fusion. Matrix localization of mCherry. Used for localization studies.
APW76	*sdhc-1(jbm1 [sdhc-1::mCherry]) III; immt-1(jbm7 [immt-1::link::GFP]) X*	*immt-1::GFP; sdhc-1::cherry*	PMID: 30887829	Ubiquitously expressed mCherry complex II fusion. Intermembrane space localization of mCherry. Used for localization studies.
APW111	*nuo-1(jbm14 [nuo-1::link::mCherry) II; immt-1(jbm7 [immt-1::link::GFP]) X*	*immt-1::GFP; nuo-1::mCherry*	this study	Ubiquitously expressed mCherry complex I fusion. Used for localization studies.
GA184	*sod-2(gk257) I*	*sod-2*	CGC	Mitochondrial superoxide dismutase mutant
GA186	*sod-3(tm760) X*	*sod-3*	CGC	Mitochondrial superoxide dismutase mutant
GA480	*sod-2(gk257) I; sod-3(tm760) X*	*sod-2;sod-3*	CGC	Mitochondrial superoxide dismutase double mutant

APW266	*sod-2(gk257) I; nuo-1(jbm16 [nuo-1::link::SuperNova]) II; sod-3(tm760) X*	*nuo-1::SuperNova; sod-2; sod-3*	this study	Complex I light-activated ROS CRISPR strain (APW92) crossed into mitochondrial superoxide dismutase double mutant (GA480).
APW271	*sod-2(gk257) I; nuo-1(jbm16 [nuo-1::link::SuperNova]) II*	*nuo-1::SuperNova; sod-2*	this study	Complex I light-activated ROS CRISPR strain (APW92) crossed into mitochondrial superoxide dismutase mutant (GA184).
APW259	*nuo-1(jbm16 [nuo-1::link::SuperNova]) II; sod-3(tm760) X*	*nuo-1::SuperNova; sod-3*	this study	Complex I light-activated ROS CRISPR strain (APW92) crossed into mitochondrial superoxide dismutase mutant (GA186).
CW152	*nduf-2.1(fc21) X*	*nduf-2.1(fc21)*	CGC	Complex I mutant. Also known as *gas-1(fc21)*
APW269	*nduf-2.1(jbm49 [nduf-2.1 C366S]) X*	*nduf-2.1 C366S*	this study	Complex I subunit mutant C366S
APW285	*nduf-2.1(jbm50 [nduf-2.1 C366D]) X*	*nduf-2.1 C366D line 1*	this study	Complex I subunit mutant C366D
APW286	*nduf-2.1(jbm51 [nduf-2.1 C366D]) X*	*nduf-2.1 C366D line 2*	this study	Complex I subunit mutant C366D
APW268	*nuo-1(jbm16 [nuo-1::link::SuperNova]) II,nduf-2.1(jbm48 [nduf-2.1 C366S])X*	*nuo-1::SuperNova nduf-2.1 C366S*	this study	Complex I light-activated ROS CRISPR strain (APW92) with complex I subunit mutant C366S.
APW281	*nuo-1(jbm16 [nuo-1::link::SuperNova]) II*<br><br>*jbmEx120[pNG1(Peft-3::2xCOX8a::HyPer7) 100 ng/µL]*	*nuo-1::SuperNova;* matrix-HyPer	this study	Complex I light-activated ROS CRISPR strain (APW92) with matrix-HyPer7 extrachromosomal array.
APW283	*WT*<br><br>*jbmEx122[pNG1(Peft-3::2xCOX8a::HyPer7) 100 ng/µL]*	N2;<br><br>matrix-HyPer	this study	N2 with matrix-HyPer7 extrachromosomal array.

TK22	*mev-1(kn1) III*	*mev-1(kn1)*	CGC	Complex II mutant. Also known as *sdhc-1(kn1)*.
APW120	*ucr-2.3(jbm24 [ucr-2.3::link::mCherry]) III; immt-1(jbm7 [immt-1::link::GFP]) X*	*immt-1::GFP; ucr-2.3::mCherry*	this study	Ubiquitously expressed mCherry complex III fusion. Used for localization studies
APW226	*ucr-11(jbm28 [ucr-11::link::mCherry]) III, immt-1(jbm7 [immt-1::link::GFP]) X*	*immt-1::GFP; ucr-11::mCherry*	this study	Ubiquitously expressed mCherry complex III fusion. Used for localization studies
APW319	*nuo-1(jbm16 [nuo-1::link::SuperNova]) II; immt-1(jbm7 [immt-1::link::GFP]) X*	*immt-I::GFP; nuo-1::SuperNova*	this study	Ubiquitously expressed SuperNova complex I fusion. Used for localization studies
APW125	*ucr-2.3(jbm21 [ucr-2.3::link::SuperNova]) III*	*ucr-2.3::SuperNova*	this study	SuperNova complex III fusion
APW146	*ucr-11(jbm30 [ucr-11::link::SuperNova]) III*	*ucr-11::SuperNova*	this study	SuperNova complex III fusion

Supplementary Table 2. List of repair templates used in CRISPR-Homology Directed Repair editing.

Strain	Repair template primers	Template	Product
APW92	FWD:GTTTTGGCCGAGCAGGGAGCCAAGCAGATCAGTCAAGGAGCATCGGGAGCCTCAGGAGCATCGATGGGTTCAGAGGTCGGCCC<br>REV:TTAAAAAGTCAGCGCTACAAACACTAAAACGGCCTTTAATCCTCGTCGCTACCGATG	pB RSET SuperNova	SuperNova
APW111	FWD:GTTTTGGCCGAGCAGGGAGCCAAGCAGATCAGTCAAGGAGCATCGGGAGCCTCAGGAGCATCGATGGTCTCAAAGGGTGAAGA<br>REV:TTAAAAAGTCAGCGCTACAAACACTAAAACGGCCTCTACTTATACAATTCATCCATGCC	pCFJ90	mCherry
APW269	GTGATCGTCGACCTTGATTTCTCCGGCTGGCATCTTATTAAGAGACTGATGCACAATGTTCAACGACTGTCTCATCTCCTC	n/a	C366S edit
APW285	GTGATCGTCGACCTTGATTTCTCCGGCTGGCATCTTATTAAGATCCTGATGCACAATGTTCAACGACTGTCTCATCTCCTC	n/a	C366D edit
APW120	FWD:ATATGGAAATTACGGAAGAATCCCATATGCCGACGAGCTTGGAGCATCGGGAGCCTCAGGAGCATCGATGGTCTCAAAGGGTGAAGA<br>REV:TAGCACAAAAAGTTGACAAAAACGAATTTAAATTACTTATACAATTCATCCATGCCAC	pCFJ90	mCherry
APW226	FWD:AGTACATCCCACTCTGGAACAAGCGTTACGTCGAAGGAGCATCGGGAGCCTCAGGAGCATCGATGGTCTCAAAGGGTGAAGA<br>REV:TTCTAAAAAAGTCAATCAACGATCGGAATAGGTGTCGATTATTTAATCTACTTATACAATTCATCCATGCCAC	pCFJ90	mCherry
APW125	FWD:ATATGGAAATTACGGAAGAATCCCATATGCCGACGAGCTTGGAGCATCGGGAGCCTCAGGAGCATCGATGGGTTCAGAGGTCGGCCC<br>REV:TAGCACAAAAAGTTGACAAAAACGAATTTAAATTAATCCTCGTCGCTACCGATG	pB RSET SuperNova	SuperNova
APW146	FWD:AGTACATCCCACTCTGGAACAAGCGTTACGTCGAAGGAGCATCGGGAGCCTCAGGAGCATCGATGGGTTCAGAGGTCGGCCC	pB RSET SuperNova	SuperNova

REV:TTCTAAAAAAGTCAATCAACGATCGGAATAGG
TGTCGATTATTTAATCTAATCCTCGTCGCTACCGAT
G

Supplementary Table 3. List of crRNA for CRISPR-Homology Directed Repair editing

**Gene Target**	**crRNA target sequence**
nuo-1	*CCAAGCAGATCAGTCAATGA*
nduf-2.1	*CTCCGGCTGGCATCTTGTTG*
ucr-11	*AACGATCGGAATAGGTGTTG*
ucr-2.3	*GAATTTAAATTAAAGCTCGT*
