# Supplementary material for: A reversible mitochondrial complex I thiol switch mediates hypoxic avoidance behavior in C. elegans
Source: Nat Commun. 2022 May 3;13:2403. doi: 10.1038/s41467-022-30169-y (PMC9064984; doi:10.1038/s41467-022-30169-y)
Supplement: Supplementary file 3 — Reporting Summary [file 41467_2022_30169_MOESM3_ESM.pdf]

nature research

Corresponding author(s): Dr. Andrew Wojtovich

Last updated by author(s): Mar 21, 2022

Reporting Summary

Nature Research wishes to improve the reproducibility of the work that we publish. This form provides structure for consistency and transparency in reporting. For further information on Nature Research policies, see our Editorial Policies and the Editorial Policy Checklist.

Statistics

For all statistical analyses, confirm that the following items are present in the figure legend, table legend, main text, or Methods section.

n/a	Confirmed	
☐	☒	The exact sample size (n) for each experimental group/condition, given as a discrete number and unit of measurement
☐	☒	A statement on whether measurements were taken from distinct samples or whether the same sample was measured repeatedly
☐	☒	The statistical test(s) used AND whether they are one- or two-sided<br>*Only common tests should be described solely by name; describe more complex techniques in the Methods section.*
☐	☒	A description of all covariates tested
☐	☒	A description of any assumptions or corrections, such as tests of normality and adjustment for multiple comparisons
☐	☒	A full description of the statistical parameters including central tendency (e.g. means) or other basic estimates (e.g. regression coefficient) AND variation (e.g. standard deviation) or associated estimates of uncertainty (e.g. confidence intervals)
☐	☒	For null hypothesis testing, the test statistic (e.g. F, t, r) with confidence intervals, effect sizes, degrees of freedom and P value noted<br>*Give P values as exact values whenever suitable.*
☒	☐	For Bayesian analysis, information on the choice of priors and Markov chain Monte Carlo settings
☒	☐	For hierarchical and complex designs, identification of the appropriate level for tests and full reporting of outcomes
☒	☐	Estimates of effect sizes (e.g. Cohen's d, Pearson's r), indicating how they were calculated

Our web collection on statistics for biologists contains articles on many of the points above.

Software and code

Policy information about availability of computer code

Data collection	Oxygen consumption data was collected using Oxygraph+ System (Hansatech Instruments Ltd). HPLC data was collected using Lab Solutions (Shimadzu). Images were collected using Slidebook6 software (Intelligent Imaging Innovation, Denver, CO) and FV1000. Human complex I (PDB: 6ZR2) was loaded into ProDy (v2.0) for normal mode analysis and visualized using the NMWizard plugin in VMD (v1.9.4).
Data analysis	Microsoft excel 2019, ProDy (v2.0), VMD (v1.9.4), image J (1.5.2), Prism 7 and Prism 9.

For manuscripts utilizing custom algorithms or software that are central to the research but not yet described in published literature, software must be made available to editors and reviewers. We strongly encourage code deposition in a community repository (e.g. GitHub). See the Nature Research guidelines for submitting code & software for further information.

Data

Policy information about availability of data

All manuscripts must include a data availability statement. This statement should provide the following information, where applicable:

- Accession codes, unique identifiers, or web links for publicly available datasets
- A list of figures that have associated raw data
- A description of any restrictions on data availability

Data generated with this study are available in the main text, and supplementary figures and tables. Previously published databases and accession codes used in the study (e.g. PDB: 6ZR2) are listed in the main text.

April 2020

Field-specific reporting

Please select the one below that is the best fit for your research. If you are not sure, read the appropriate sections before making your selection.

☒ Life sciences ☐ Behavioural & social sciences ☐ Ecological, evolutionary & environmental sciences

For a reference copy of the document with all sections, see nature.com/documents/nr-reporting-summary-flat.pdf

Life sciences study design

All studies must disclose on these points even when the disclosure is negative.

Sample size	Hypoxia-, light-, and toxin-mediated behavioral study sample sizes were calculated using a power analysis (StatMate, GraphPad). Localization, ROS measurements, bioenergetic, and hypoxia reoxygenation study sample sizes were determined using previous publications (Berry 2020a, Berry 2020b, Trewin 2019).
Data exclusions	No data were excluded from the analyses.
Replication	Each experiment is composed of at least three technical replicates. Each technical replicate can be composed of multiple animals or trials. The specific number of replicates and how an "N" is defined is described in the figure legends for each experiment.
Randomization	Experimental groups were determined for each genotype and animals were randomly assigned to treatment groups.
Blinding	The experimenter was blinded to the genotype or condition.

Reporting for specific materials, systems and methods

We require information from authors about some types of materials, experimental systems and methods used in many studies. Here, indicate whether each material, system or method listed is relevant to your study. If you are not sure if a list item applies to your research, read the appropriate section before selecting a response.

Materials & experimental systems

n/a	Involved in the study	
☐	☒	Antibodies
☒	☐	Eukaryotic cell lines
☒	☐	Palaeontology and archaeology
☐	☒	Animals and other organisms
☒	☐	Human research participants
☒	☐	Clinical data
☒	☐	Dual use research of concern

Methods

n/a	Involved in the study	
☒	☐	ChIP-seq
☒	☐	Flow cytometry
☒	☐	MRI-based neuroimaging

Antibodies

Antibodies used	Primary Antibody: rabbit anti-KillerRed: Evrogen, Cat#AB961, Lot#96101240513, 1:1000 dilution. Note: Supernova is the monomeric version of tdKillerRed.<br><br>Fluorescent secondary antibody: Goat-anti-rabbit, Bio-Rad, Starbright 700 cat# 12004158, lot #64247470. 1:5000 dilution.
Validation	Validation of the primary antibody is noted on the manufacturer's website and shows the selectivity of the antibody for KillerRed and and not other proteins. Additionally, using transgenic animals expressing the protein, we demonstrate the selectivity of the antibody in Supplemental Figures 3 and 9 and in PubMed ID: 30887829.

Animals and other organisms

Policy information about studies involving animals; ARRIVE guidelines recommended for reporting animal research

Laboratory animals	Caenorhabditis elegans were used in this study and some strains were provided by the Caenorhabditis Genetics Center. This study used L4, Day 1, Day 3 and Day 5 hermaphrodites and a complete list of strains is provided in Table 1.
Wild animals	The study did not involve wild animals.
Field-collected samples	The study did not involve field-collected samples.
Ethics oversight	C. elegans is an invertebrate. Experiments were approved by the University of Rochester Institutional Biosafety Committee.

Note that full information on the approval of the study protocol must also be provided in the manuscript.
